# Risk assessment of rectal anastomotic leakage (RAREAL) after DIXON in non-emergency patients with rectal cancer

**DOI:** 10.1186/s12876-023-02982-2

**Published:** 2023-10-03

**Authors:** Xue-Cong Zheng, Jin-Bo Su, Jin-Jie Zheng

**Affiliations:** 1 Department of General Surgery, the Second Affiliated Hospital of Fujian Medical University, Quanzhou, Fujian 362000 China; 2 Endocrine Department, Quanzhou First Hospital Affiliated to Fujian Medical University, Quanzhou, Fujian 362000 China

**Keywords:** Risk assessment of rectal anastomotic leak (RAREAL), Anastomotic leakage (AL), Rectal cancer, DIXON

## Abstract

**Background:**

The routine establishment of a diverting stoma (DS) remains controversial in every patient undergoing Dixon operation. We aimed to establish a model for the risk assessment of rectal anastomotic leak (RAREAL) after Dixon in non-emergency patients with rectal cancer, using routinely available variables, by which surgeons could individualize their approach to DS.

**Methods:**

323 patients who underwent Dixon operation for rectal cancer from January 2015 to December 2018 were taken as the model group for retrospective study. Univariable and multivariable logistic regression analysis was used to determine the independent risk factors associated with anastomotic leakage. We constructed the RAREAL model. 150 patients who underwent Dixon operation due to rectal cancer from January 2019 to December 2020 were collected according to the uniform criteria as a validation group to validate the RAREAL model.

**Results:**

In the model group, multivariable analysis identified the following variables as independent risk factors for AL: HbA1c (odds ratio (OR) = 4.107; *P* = 0.044), Left colic artery (LCA) non preservation (OR = 4.360; *P* = 0.026), Tumor distance from the anal margin (TD) (OR = 6.373; *P* = 0.002). In the model group, the area under the curve (AUC) of the receiver operating characteristic (ROC) for evaluating AL with RAREAL was 0.733, and when RAREAL score = 2.5, its sensitivity, specificity and Youden index were 0.385, 0.973, 0.358, respectively. The AUC was 0.722 in the validation group and its sensitivity and specificity were 0.333 and 0.985, respectively, when RAREAL score = 2.5.

**Conclusion:**

The RAREAL score can be used to assess the risk of AL after Dixon operation for rectal cancer, and prophylactic DS should be proactively done when the score is greater than 2.5.

## Background

Low anterior resection with preservation of the anal sphincter (Dixon operation) is an increasingly common procedure for rectal cancer surgery. Anastomotic leakage (AL) is a major and serious surgical complication after anterior resection of rectal cancer, which continues to occur despite advances in surgical techniques and treatments. The reported incidence of AL in rectal surgery ranges from 3–19% [1]. AL is associated with prolonged hospital stay, increased medical costs, and higher morbidity and mortality in a short period of time. AL also promotes pelvic tumor recurrence and reduces overall survival [2].

Given the serious consequences of AL, most surgeons prefer prophylactic diverting stoma (DS) for high-risk patients with AL. But many of them may not develop AL even without a prophylactic DS, which would put the patient at risk of DS related complications and second surgical closure. Studies have shown that DS significantly delays postoperative recovery of patients. DS and stoma closure can lead to severe complications such as intestinal leak, ileus, intestinal perforation, intra-abdominal infection, disturbance of water electrolyte balance and renal dysfunction, as well as impair patient quality of life [3, 4]. And there may be increased spending on health care funds in these situations, which surgeons need to consider carefully.

Therefore, rapid and accurate prediction of AL assessment is essential for early treatment and DS in Dixon patients, while avoiding unnecessary invasive procedures in low-risk patients. Our aim was to identify preoperative and intraoperative risk factors using simple and commonly used variables. These risk factors were then used to establish a risk assessment of rectal anastomotic Leak (RAREAL) model after DIXON surgery for rectal cancer, which was used to assess the risk of developing AL. We used this risk assessment model to help surgeons decide whether to perform a protective DS when performing sphincter-preserving surgery for rectal cancer.

## Materials and methods

### Patients and study design

We retrieved the electronic medical records of the Second Affiliated Hospital of Fujian Medical University between January 2015 and December 2020, and selected the information of 721 hospitalized patients who had undergone Dixon surgery for rectal cancer who were hospitalized during this period. Exclusion criteria: patients with intestinal obstruction (47 cases), patients with rectal perforation (6 cases), patients with distant metastasis (38 cases), patients with incomplete hospitalization data (26 cases), and patients with prophylactic diverting stoma (131 cases). A total of 473 consecutive patients who had undergone DIXON for rectal cancer were screened and collected. Signed informed consent was obtained from all study subjects. This study was approved by the ethics committee of the Second Affiliated Hospital of Fujian Medical University (2,022,208).

**Fig. 1:**
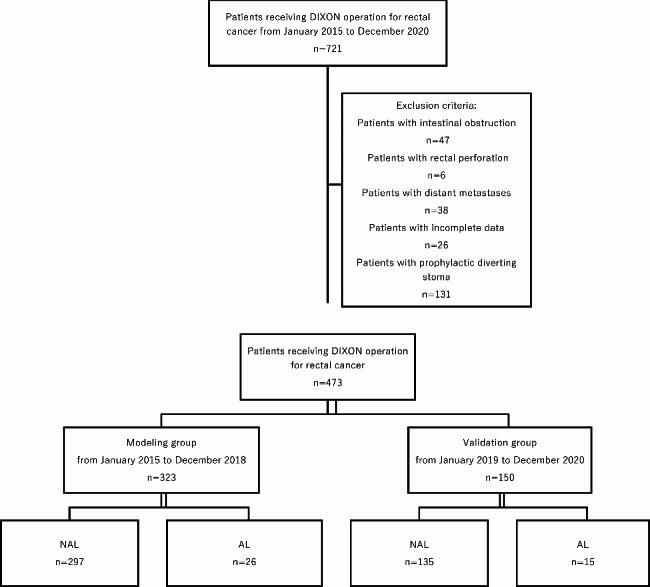
Flowchart of experimental design in our study

A total of 323 hospitalized patients who underwent Dixon surgery for rectal cancer from January 2015 to December 2018 were used as the modeling group, which were divided into NAL group (297 patients) and AL group (26 patients) according to whether AL occurred after surgery. A total of 150 hospitalized patients who underwent DIXON surgery for rectal cancer from January 2019 to December 2020 were selected as the validation group, including the NAL group (135 cases) and the AL group (15 cases) (Fig. 1).

The investigated variables were age, gender, smoking, Alcohol excess, body mass index (BMI), Previous history of malignancy, hypertension, preoperative chemoradiotherapy (PCT), diabetes mellitus, HbA1c (glycated hemoglobin), American Society of Anesthesiologists physical status classification (ASA ), operation time, intraoperative blood loss, left colic artery (LCA) preservation, tumor distance from the anal margin (TD), number of staples, surgical approach, pathological T stage, pathological N stage, TNM stage, and tumor histological differentiation.

Each patient underwent bowel preparation by oral polyethylene glycol electrolyte solution combined with prophylactic oral enteric antibiotics on the first preoperative day. All surgeries were performed by experienced and skilled colorectal surgeons. According to the criteria of the learning curve of laparoscopic colorectal cancer resection, after 40 laparoscopic colorectal cancer surgeries were carried out, the operating technique of the operating surgeon could achieve a degree of comparative stability [5, 6]. We considered surgeons who carry out more than 40 laparoscopic colorectal cancer procedures to be experienced and skilled colorectal surgeons. The principles of total mesorectal excision (TME) were strictly followed for both open and laparoscopic procedures. For transection and anastomosis of the rectum, linear cutting closure device was used for rectal resection, and circular stapler was used for rectal anastomosis. In each patient with an anastomosis for rectal resection, an ECHELON linear cut closure device was used for rectal resections, and an Ethicon Endo-surgery circular stapler was used for rectal anastomoses. The circular stapler was a double-rows. In recent years, intraoperative indocyanine green (ICG) angiography has been introduced into clinical practice to provide useful information on intraoperative vascular perfusion. However, according to some studies, it does not significantly reduce anastomotic leakage rates [7, 8]. There are still some drawbacks to the technique of intraoperative ICG angiography evaluation. One drawback is the optimal dosage and timing of ICG prior to evaluation. The vascular system has a fast washout time, and an accurate injection protocol is still to be discovered. Another drawback is the lack of strict analysis methods to objectively quantify signal intensity, and the evaluation of images still depends on the surgeon’s judgment. It is currently unclear what precise blood flow rate is required to ensure satisfactory healing of intestinal tissues [7]. Intraoperative air leak test (ALT) is a widely accepted measure to prevent AL [9, 10]. After anastomosis, the ALT was performed on all patients. When the leak test was positive, the anastomosis was repaired by suturing until a negative result was obtained. After anastomosis, the pelvic cavity and abdominal cavity were irrigated with a large amount of distilled water to clean, and a rubber drain was routinely left in place next to the rectal anastomosis in the pelvic cavity.

A multidisciplinary team (MDT) of physicians with independent diagnostic and therapeutic capabilities, mainly from colorectal surgery and anesthesiology departments, including cardiovascular medicine, respiratory medicine, endocrinology, neurology, hematology, Nephrology, nutrition and other related clinical departments, was established. Each rectal cancer patient was consulted by the MDT team. If the patient had diseases for which no preoperative normative treatment has been administered such as: severe hypertension, arrhythmia, bronchial asthma attack, severe chronic bronchitis, acute exacerbation of emphysema, pneumonia, poorly controlled severe hyperglycemia, severe thyroid dysfunction, brain dysfunction, uncontrolled coagulopathy, severe anemia, severe electrolyte imbalance, malnutrition, psychological disorders, and so on, the MDT team would administer optimized treatments to the patient, and then the patient would undergo surgical treatments at a limited period. All patients received adequate nutrition, anti-infection and other symptomatic treatment after operation.

### Definition of AL

**Fig. 2:**
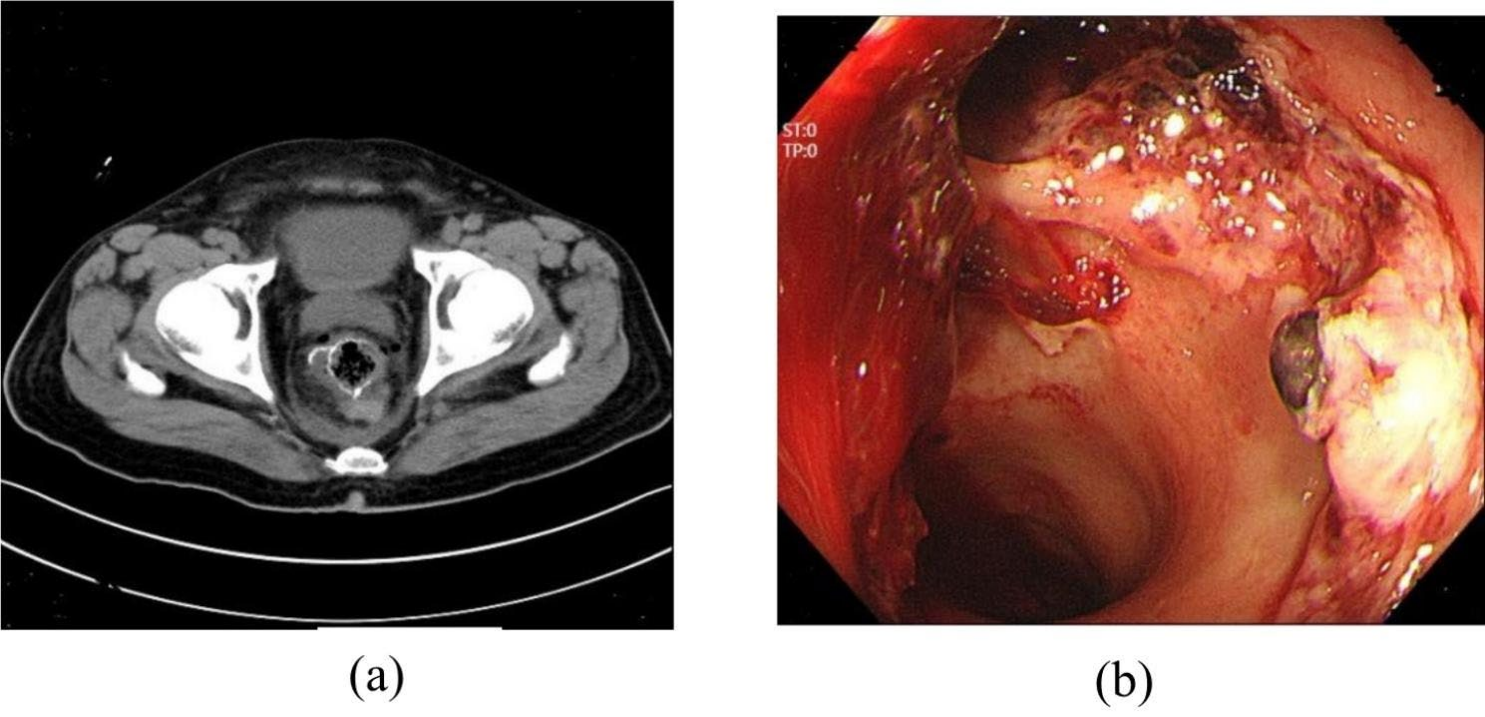
Image of AL. **(a)** Computed tomography scan of the pelvis: air bubbles and fluid around the rectal anastomosis; **(b)** Colonoscopy: rectal anastomotic defect

Clinical diagnostic criteria for AL [11]: clinical signs of AL were considered if the pelvic drain was draining pus or stool and there were suspicious symptoms of peritonitis, including abdominal pain, tenderness, fever, tachycardia, or severe inflammation on blood tests. AL was further confirmed by CT scan or colonoscopy. Positive CT scan: abscess and effusion or air bubbles around the anastomosis; Positive colonoscopy: the rectal cavity communicates with the extracavity (Fig. 2). Enema with contrast agent was not routinely performed in our department, and asymptomatic AL was not considered. All rectal cancers were confirmed as primary rectal adenocarcinomas by preoperative colonoscopy as well as postoperative pathology.

### Statistical analysis

Statistical analysis was performed using SPSS 21.0 software. Normally distributed measurement data are represented by $\stackrel{-}{x}\pm s$, and skewed distribution data are represented by median (first and third quartiles) [M (P25, P75)]. Enumeration data were expressed as the number of cases (%). The t test, χ²test and Mann-Whitney U test were used for statistical analysis of the two groups of data in the NAL group and the AL group. Multivariable Logistic regression analysis was used to obtain the risk factors of the AL group, and the partial regression coefficient was rounded into the score of the corresponding risk factor index. Risk assessment of rectal anastomotic leak (RAREAL) score was used to express the risk of AL group. The area under the curve (AUC) of the receiver operating characteristic (ROC) and the Youden index were used to evaluate the RAREAL score. The maximum Youden index obtained corresponds to the sensitivity and specificity of RAREAL. The RAREAL score was performed on the patients in the modeling group, the ROC curve was used for analysis, and the Z test was used to evaluate the consistency of the ROC curve between the modeling group and the validation group. *P*<0.05 was considered to be statistically significant.

## Result

### Analysis of clinical data

A total of 473 patients who underwent DIXON surgery for rectal cancer were retrospectively collected. The incidence of AL was 8.7% (41/473), of which the incidence of AL in the model group and AL in the validation group were 8.0% (26/323) and 10.0% (15/150), respectively. There were no statistically significant differences between the two groups (model and validation) as shown in Table 1.

**Table 1:** characteristics of patients in the model group and the validation group

Factors	Model (n = 323)		Validation (n = 150)		*P*		
Patient related factors							
Age(years, $\stackrel{-}{x}\pm s$)	59.5 ± 11.6		59.3 ± 10.8		0.829		
Gender, n = No (%)					0.442		
Female	111(34.4%)		57(38.0%)				
Male	212(65.6%)		93(62.0%)				
AL					0.483		
Yes	26(8.0%)		15(10.0%)				
No	297(92.0%)		135(90.0%)				
Smoking, n = No (%)					0.201		
Yes	85(26.3%)		48(32.0%)				
No	238(73.7%)		102(68.0%)				
Alcohol excess, n = No (%)					0.296		
Yes	76(23.5%)		42(28.0%)				
No	247(76.5%)		108(72.0%)				
Previous history of malignancy, n = No (%)					0.704		
Yes		37(11.5%)		19(12.7%)			
No		286(88.5%)		131(87.3%)			
BMI(kg/m^2^, $\stackrel{-}{x}\pm s$)	22.5 ± 2.7		22.8 ± 2.6		0.335		
HbA1c, n = No (%)							
≤ 6.3%	304(94.1%)		141(94.0%)		0.960		
> 6.3%	19(5.9%)		9(6.0%)				
Diabetes mellitus, n = No (%)					0.936		
Yes	70(21.7%)		33(22.0%)				
No	253(78.3%)		117(78.0%)				
Hypertension, n = No (%)					0.632		
Yes	104(32.2%)		45(30.0%)				
No	219(67.8%)		105(70.0%)				
PCT, n = No (%)					0.338		
Yes	49(15.2%)		28(18.7%)				
No	274(84.8%)		122(81.3%)				
ASA grade, n = No (%)					0.547		
3	38(11.8%)		13(8.7%)				
2	98(30.3%)		50(33.3%)				
1	187(57.9%)		87(58.0%)				
Surgical related factors							
Operation time(min)					0.162		
Median(IQR)	170(155, 190)		180(155, 210)				
Intraoperative blood loss (ml)					0.836		
Median(IQR)	40 (40,50)		40 (40,50)				
LCA preservation, n = No (%)					0.435		
Yes	280(86.7%)		126(84.0%)				
No	43(13.3%)		24(16.0%)				
Number of staples fired, n = No (%)					0.192		
1 or 2	262(81.1%)		129(86.0%)				
>2	61(18.9%)		21(14.0%)				
Surgical approach, n = No (%)					0.212		
Laparoscopic	270(83.6%)		132(88.0%)				
Open	53(16.4%)		18(12.0%)				
Tumor related factors							
TD, n = No (%)					0.709		
≥ 7 cm	161(49.8%)		72(48.0%)				
< 7 cm	162(50.2%)		78(52.0%)				
Pathological T stage, n = No (%)					0.136		
Tis, T1, T2,	121(37.5%)		67(44.7%)				
T3 and T4	202(62.5%)		83(55.3%)				
Pathological N stage, n = No (%)					0.905		
N0	90(27.9%)		41(27.3%)				
N1 and N2	233(72.1%)		109(72.7%)				
TNM stage, n = No (%)					0.126		
I	35(10.8%)		10(6.7%)				
II	129(39.9%)		73(48.7%)				
III	159(49.2%)		67(44.7%)				
Tumor histological differentiation, n = No (%)					0.419		
High	126(39.0%)		55(36.7%)				
Moderate	97(30.0%)		54(36.0%)				
Low	100(31.0%)		41(27.3%)				

AL anastomotic leakage, BMI body mass index, HbA1c glycated hemoglobin, PCT preoperative chemoradiotherapy, ASA American Society of Anesthesiologists physical status classification, IQR interquartile range, LCA left colic artery, TD tumor distance from the anal margin

### Analysis of patient related factors in the model group

Compared with the NAL group, there were more male patients (*P* = 0.034) and PCT (*P* = 0.021) in the AL group. Patients with HbA1c (> 6.3%) also had significantly higher AL after surgery (*P* = 0.032). There was no significant difference in age, smoking, Alcohol excess, BMI, Previous history of malignancy, Diabetes mellitus, hypertension and ASA between AL group and NAL group.

### Analysis of surgical related factors in model group

There were more patients with LCA non preservation (*P* = 0.033) and with more than 2 staples (*P* = 0.016) in the AL group compared with the NAL group. There was no significant difference in operation time, intraoperative blood loss, surgical approach and DS between the AL group and the NAL group.

### Analysis of tumor related factors in the model group

Compared with the NAL group, the AL group had more patients with TD (< 7 cm) (*P* = 0.001). There was no significant difference in pathological T stage, pathological N stage, TNM stage and tumor histological differentiation.

The clinical information and results of the univariable analysis of the patients in the model group are shown in Table 2.

**Table 2:** characteristics of patients in the model group

Factors	AL (n = 26)		NAL (n = 297)		*P*		
Patient related factors							
Age(years, $\stackrel{-}{x}\pm s$)	61.9 ± 7.6		59.3 ± 11.9		0.276		
Gender, n = No (%)					0.034		
Female	4(15.4%)		107(36.0%)				
Male	22(84.6%)		190(64.0%)				
Smoking, n = No (%)					0.942		
Yes	7(26.9%)		78(26.3%)				
No	19(73.1%)		219(73.7%)				
Alcohol excess, n = No (%)					0.364		
Yes	8(30.8%)		68(22.9%)				
No	18(69.2%)		229(77.1%)				
Previous history of malignancy, n = No (%)					0.530		
Yes		2(7.7%)		35(11.8%)			
No		24(92.3%)		262(88.2%)			
BMI(kg/m^2^, $\stackrel{-}{x}\pm s$)	22.2 ± 2.1		22.6 ± 2.7		0.473		
HbA1c, n = No (%)					0.032		
≤ 6.3%	22(84.6%)		282(94.9%)				
> 6.3%	4(15.4%)		15(5.1%)				
Diabetes mellitus, n = No (%)					0.417		
Yes	4(15.4%)		66(22.2%)				
No	22(84.6%)		231(77.8%)				
Hypertension, n = No (%)					0.783		
Yes	9(34.6%)		95(32.0%)				
No	17(65.4%)		202(68.0%)				
PCT, n = No (%)					0.021		
Yes	8(30.8%)		41(13.8%)				
No	18(69.2%)		256(86.2%)				
ASA grade, n = No (%)					0.916		
3	3(11.5%)		35(11.8%)				
2	7(26.9%)		91(30.6%)				
1	16(61.5%)		171(57.6%)				
Surgical related factors							
Operation time(min)					0.265		
Median(IQR)	180(150.0, 213.4)		170(155.0, 190.0)				
Intraoperative blood loss (ml)					0.572		
Median(IQR)	45.0 (27.5,72.5)		40.0 (40.0,50.0)				
LCA preservation, n = No (%)					0.033		
Yes	19(73.1%)		261(87.9%)				
No	7(26.9%)		36(12.1%)				
Number of staples fired, n = No (%)					0.016		
1 or 2	16(61.5%)		246(82.8%)				
>2	10(38.5%)		51(17.2%)				
Surgical approach, n = No (%)					0.685		
Laparoscopic	21(80.8%)		249(83.8%)				
Open	5(19.2%)		48(16.2%)				
Tumor related factors							
TD, n = No (%)					0.001		
≥ 7 cm	5(19.2%)		156(52.5%)				
< 7 cm	21(80.8%)		141(47.5%)				
Pathological T stage, n = No (%)					0.114		
Tis, T1, T2,	6(23.1%)		115(38.7%)				
T3 and T4	20(76.9%)		182(61.3%)				
Pathological N stage, n = No (%)					0.139		
N0	4(15.4%)		86(29.0%)				
N1 and N2	22(84.6%)		211(71.0%)				
TNM stage, n = No (%)					0.172		
I	3(11.5%)		32(10.8%)				
II	6(23.1%)		123(41.4%)				
III	17(65.4%)		142(47.8%)				
Tumor histological differentiation, n = No (%)					0.402		
High	12(46.2%)		114(38.4%)				
Moderate	9(34.6%)		88(29.6%)				
Low	5(19.2%)		95(32.0%)				

AL anastomotic leakage, NAL non anastomotic leakage, BMI body mass index, HbA1c glycated hemoglobin, PCT preoperative chemoradiotherapy, ASA American Society of Anesthesiologists physical status classification, IQR interquartile range, LCA left colic artery, TD tumor distance from the anal margin

### Information for validation group

The incidence of AL in 150 patients undergoing DIXON surgery for rectal cancer was 10.0% (15/150). Patients with TD (< 7 cm) were 52.0% (78/150) which was slightly higher than 50.2% (162/323) of the model group. Among them, there are 135 cases of NAL and 15 cases of AL. The information of the patients in the validation group was shown in the Table 3.

**Table 3:** characteristics of patients in the validation group

Factors	AL(n = 15)	NAL(n = 135)	P
Patient related factors			
Age(years, $\stackrel{-}{x}\pm s$)	63.3 ± 9.7	58.6 ± 10.8	0.133
Gender, n = No (%)			0.130
Female	3(20.0%)	54(40.0%)	
Male	12(80.0%)	81(60.0%)	
Smoking, n = No (%)			0.907
Yes	5(33.3%)	43(31.9%)	
No	10(66.7%)	92(68.1%)	
Alcohol excess, n = No (%)			0.904
Yes	4(26.7%)	38(28.1%)	
No	11(73.3%)	97(71.9%)	
Previous history of malignancy, n = No (%)			0.461
Yes	1(6.7%)	18(13.3%)	
No	14(93.3%)	117(86.7%)	
BMI(kg/m^2^, $\stackrel{-}{x}\pm s$)	22.4 ± 2.2	22.8 ± 2.6	0.473
HbA1c, n = No (%)			0.207
≤ 6.3%	13(86.7%)	128(94.8%)	
> 6.3%	2(13.3%)	7(5.2%)	
Diabetes mellitus, n = No (%)			0.393
Yes	2(13.3%)	31(23.0%)	
No	13(86.7%)	104(77.0%)	
Hypertension, n = No (%)			0.373
Yes	6(40.0%)	39(28.9%)	
No	9(60.0%)	96(71.1%)	
PCT, n = No (%)			0.402
Yes	4(26.7%)	24(17.8%)	
No	11(73.3%)	111(82.2%)	
ASA grade, n = No (%)			0.957
3	1(6.7%)	12(8.9%)	
2	5(33.3%)	45(33.3%)	
1	9(57.8%)	78(57.8%)	
Surgical related factors			
Operation time (min)			0.478
Median(IQR)	180(175,210)	180(155,210)	
Intraoperative blood loss (ml)			0.894
Median(IQR)	40(20,80)	40(40,50)	
LCA preservation, n = No (%)			0.235
Yes	11(73.3%)	115(85.2%)	
No	4(26.7%)	20(14.8%)	
Number of staples fired, n = No (%)			0.136
1 or 2	11(73.3%)	118(87.4%)	
>2	4(26.7%)	17(12.6%)	
Surgical approach, n = No (%)			0.867
Laparoscopic	13(86.7%)	119(88.1%)	
Open	2(13.3%)	16(11.9%)	
Tumor related factors			
TD, n = No (%)			0.022
≥ 7 cm	3(20.0%)	69(51.1%)	
< 7 cm	12(80.0%)	66(48.9%)	
Pathological T stage, n = No (%)			0.352
Tis, T1, T2,	5(33.3%)	62(45.9%)	
T3 and T4	10(66.7%)	73(54.1%)	
Pathological N stage, n = No (%)			0.329
N0	2(13.3%)	39(28.9%)	
N1 and N2	13(86.7%)	96(71.1%)	
TNM stage, n = No (%)			0.925
I	1(6.7%)	9(6.7%)	
II	8(53.3%)	65(48.1%)	
III	6(40.0%)	61(45.2%)	
Tumor histological differentiation, n = No (%)			0.288
High	8(53.3%)	47(34.8%)	
Moderate	5(33.3%)	49(36.3%)	
Low	2(13.3%)	39(28.9%)	

AL anastomotic leakage, NAL non anastomotic leakage, BMI body mass index, HbA1c glycated hemoglobin, PCT preoperative chemoradiotherapy, ASA American Society of Anesthesiologists physical status classification, IQR interquartile range, LCA left colic artery, TD tumor distance from the anal margin

### Construction of RAREAL model

#### Transformations that convert continuous variables to categorical variables

For the convenience of constructing the RAREAL model, the glycated hemoglobin (HbA1c) variable, which is a continuous variable, was converted into a dichotomous variable (> 6.3%, ≤ 6.3%)[> 45mmol/mol,≤45mmol/mol] according to the clinical test reference range of HbA1c (3.9-6.3%)[30 mmol/mol − 45mmol/mol] .

#### Model building

AL after DIXON operation for rectal cancer was used as the dependent variable, and age, gender, smoking, alcohol excess, BMI, Previous history of malignancy, hypertension, PCT, diabetes mellitus, HbA1c, ASA, operation time, intraoperative blood loss, LCA preservation, number of staples, surgical approach, TD, pathological T stage, pathological N stage, TNM stage, and tumor histological differentiation were used as independent variables. Univariable analysis showed that male (*P* = 0.034), HbA1c (> 6.3%) (*P* = 0.032), PCT (P = 0.021), LCA non preservation (*P* = 0.033), TD (< 7 cm) (*P* = 0.001) and more than 2 staples fired during surgery (*P* = 0.016) were risk factors for AL. Further logistic regression analysis found that HbA1c (> 6.3%) (odds ratio (OR) 4.107; 95% confidence interval (CI) 1.037–16.267; *P* = 0.044), LCA non preservation (OR 4.360; 95% CI 1.190-15.976; *P* = 0.026), TD (< 7 cm) (OR 6.373; 95% CI 2.027–20.031; *P* = 0.002) were independent of AL risk factors (Table 4). Multivariable equation: Y=-6.778 + 1.413 × 1 + 1.472 × 2 + 1.852 × 3 (Y: AL, X1: HbA1c (> 6.3%), X2: LCA non preservation, X3: TD (< 7 cm)).

**Table 4:** Multivariable logistic regression analysis

Factor	B	P值	OR	95% CI
Male	0.887	0.132	2.429	0.766–7.705
HbA1c (> 6.3%)	1.413	0.044	4.107	1.037–16.267
PCT	0.272	0.363	0.795	0.098–5.929
LCA non preservation	1.472	0.026	4.360	1.190-15.976
TD	1.852	0.002	6.373	2.027–20.031
Number of staples fired	0.986	0.306	2.680	0.405–17.720

HbA1c glycated hemoglobin, PCT preoperative chemoradiotherapy, LCA left colic artery, TD tumor distance from the anal margin

The partial regression coefficients of the equation were 1.413, 1.472, and 1.852, respectively. The partial regression coefficients were rounded to the corresponding scores of the indicators to establish the RAREAL model for AL. The score for HbA1c (> 6.3%) was 1, for LCA non preservation was 1, and for TD (< 7 cm) was 2.

### Diagnostic effect of RAREAL model analyzed by ROC curve and comparison of ROC curve of two groups

**Fig. 3:**
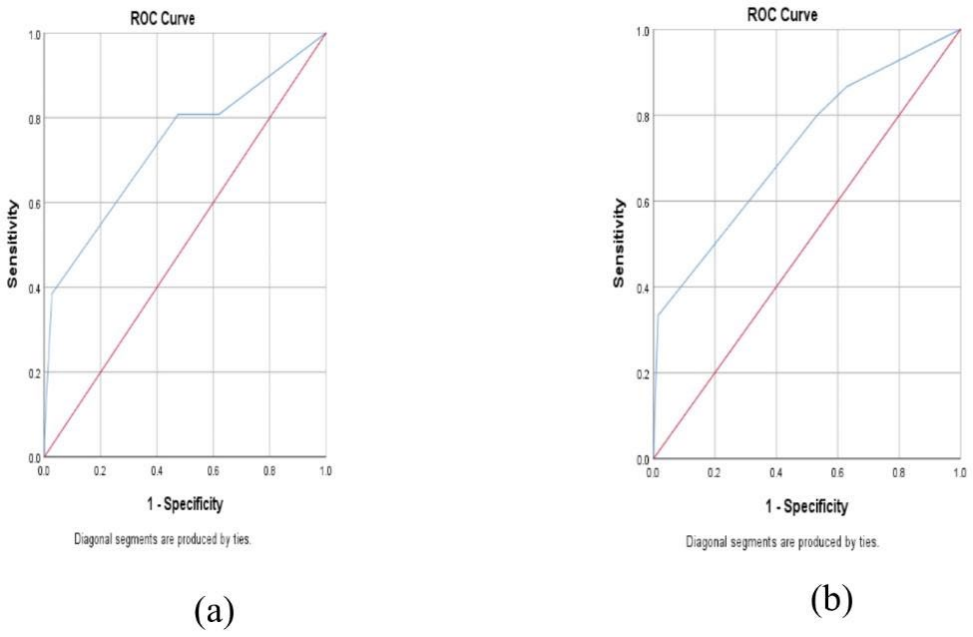
**(a)** The ROC (AUC 0.733) for the diagnosis of AL with the RAREAL score in the modeling group; **(b)** The ROC (AUC 0.722) for the diagnosis of AL with the RAREAL score in the validation group

When the maximum Youden index of the model group was 0.358, the corresponding best cut-off point of RAREAL was 2.5, the AUC (95%CI) was 0.733 (0.615–0.852), and its diagnostic sensitivity and specificity were 0.385, 0.973 (Fig. 3a). When the RAREAL score was 2.5 as the diagnostic cutoff in the validation group, the AUC (95%CI) was 0.722 (0.573–0.871), and its corresponding sensitivity and specificity were 0.333 and 0.985, respectively (Fig. 3b). There was no significant difference in the AUC of the ROC between the two groups (P = 0.9096) (Table 5).

**Table 5:** Evaluation of the consistency of ROC curve between the model group and the validation group

Group	Youden index	Best cut-off point	AUC (95%CI)	Sensitivity	Specificity	Standard error	Z	P
Model group	0.358	2.5	0.733(0.615–0.852)	0.385	0.973	0.060		
Validation group			0.722(0.573–0.871)	0.333	0.985	0.076		
							0.114	0.9096

### RAREAL scoring system

The score for HbA1c (> 6.3%) was 1, for LCA non preservation was 1, and for TD (< 7 cm) was 2 in RAREAL model. Number of patients and incidence of AL corresponding to the score values of the RAREAL scoring system (Table 6). When the score was greater than 2.5, the incidence of AL was significantly higher (58.33%). This was only a model with a small sample and was of some reference value. With the progress of research and the improvement of data, more clinical data would be included in the discussion, and the established prediction model would be closer to the actual situation.

**Table 6:** Number of patients and incidence of AL corresponding to the score values of the RAREAL scoring system

Score	Case(n = 473)	AL(n = 41)	Incidence of AL
0	170	7	4.12%
1	57	1	1.75%
2	221	18	8.14%
3	24	14	58.33%
4	1	1	100%

AL anastomotic leakage

## Discussion

AL is a major and serious surgical complication after anterior resection of rectal cancer。Many previous clinical studies have investigated many potential risk factors for AL after colorectal cancer surgery, including multiple perioperative factors [1, 12–14]; combined with relevant risk factors, a variety of predictive models for AL after colorectal cancer surgery have been established [15–18]; however, the weights used in their reported models were different, and emergency factors were taken into account in many studies. To date, no accurate risk prediction model for AL after anterior rectal resection has been established. Emergency patients did not have sufficient time to correct risk factors such as anemia, hypoalbuminemia, diabetes, hypertension, and water and electrolyte disturbances. In emergency patients, due to the rushed preoperative preparation, it is not possible to completely remove feces from the intestinal lumen, which reduces bacterial colonization. Poor bowel preparation increases the risk of complications such as AL and wound infection after surgery. Due to the intestinal dilation and the intestinal wall edema, the difficulty of intraoperative surgical procedures and dissection in emergency patients would also increase. All these factors also had a greater impact on intraoperative risk factors. These factors would also have a negative impact on cardiopulmonary function, and there would be more chances of postoperative cardiopulmonary complications, inflammatory storm, postoperative complications and AL. Many risk factors would have sufficient time to be corrected and improved in non-emergency limited-term surgery patients. For non-emergency patients with limited-term surgery, if these preoperative correctable risk factors were included in the analysis and prediction, the model construction may be biased and the performance of the model would be adversely affected, and the findings of these studies have not been further validated in other cohorts.

The patients in our study were all non-emergency surgical patients at a restricted period, with sufficient time to complete relevant preoperative tests and correct comorbidities such as hypoalbuminemia, anemia, hypertension, and hyperglycemia preoperatively, with better adjustment of the cardiopulmonary function. The study of postoperative factors affecting AL may be more real and reasonable.

Patient related factors: In this study, male, PCT, and HbA1c (> 6.3%) were risk factors for postoperative AL. Male was a risk factor for AL, possibly due to the narrower and more complex anatomy of the male pelvis, which increased the difficulty of operation and transection of the rectal bowel [14, 19]. For patients with locally advanced rectal cancer, neoadjuvant therapy has been recognized as an important treatment modality to improve clinical outcomes. Previous studies have shown that PCT generally increases infection of the perineum and perineal wounds and prolongs anastomotic healing, possibly due to compromised immune systems that fight infection and tumor immunity. In addition, PCT usually caused inflammation and edema of the anastomosis, resulting in relative anastomotic ischemia, local inflammation and tissue fibrosis, which was not conducive to anastomotic healing, increases the risk of postoperative wound infection and pelvic abscess, and increases the incidence of AL [20]. The incidence of complications and mortality after major surgery in diabetic patients were significantly higher than those in non-diabetic patients. Poor glycemic control was an important risk factor for postoperative complications in diabetic patients, especially in relation to the incidence of microvascular complications [21]. In our study, it was found that the Diabetes mellitus was not significantly related to AL after rectal cancer surgery, but high HbA1c was associated with the risk of rectal AL. Further multivariable analysis found that HbA1c (> 6.3%) was an independent risk factor for AL. HbA1c reflected average blood glucose over approximately 3 months and has strong predictive value for diabetic complications. Reducing HbA1c from 7 to 6% [53 mmol/mol to 42 mmol/mol] reduced microvascular complications of diabetes [22].

Tumor related factors and surgical related factors: In our study, it was found that LCA non preservation, low rectal cancer and more than two staples fired were the risk factors for AL. In many previous studies, the level of rectal anastomosis has been considered a risk factor for AL [23], and low rectal cancer was found to be an independent risk factor in the present study. Low rectal cancer meant that the distance from the anus of the anastomosis was less than 5 cm intraoperatively and the difficulty of performing rectal transection and rectal anastomotic techniques increased, in addition, a reduced level of anastomosis could also lead to a reduced blood supply to the anastomosis. In our study, intraoperative LCA non preservation was clearly associated with AL and was an independent risk factor. The blood supply of the anastomosis was of great importance to prevent the occurrence of AL, which has become a consensus that therapeutic surgery preserving LCA could improve the blood supply of the anastomosis and reduce the complications of the anastomosis [24, 25]. Intraoperative more than 2 staples were found to be a risk factor for AL in this study. Rectal cancer surgery was generally more difficult than colon cancer surgery. Because of the poor surgical field of view and the limited operating space of the pelvic cavity, the angle of cleavage of linear stapler cut was not well adjusted during surgery, which increased the difficulty of rectal transection, especially when low rectal resections were performed, often requiring more linear stapler. An increased number of linear stapler might cause small defects between the staples, while excessive pressing of the tissue was bound to cause local endothelial damage and secondary thrombosis affecting the local blood supply, leading to complications such as AL [13, 26].

Based on our results, a scoring model for the RAREAL (Risk Assessment of REctal Anastomotic Leak) was developed. The score for high HbA1c was 1, for LCA non preservation was 1, and for low rectal cancer was 2. The AUC of the RAREAL model was 0.733, its diagnostic sensitivity and specificity were 38.5% and 97.3%, respectively, and the AUC of the validation group was 0.722, its diagnostic sensitivity was 33.3% and specificity was 98.5%. the AUC of the ROC of the two groups was not significantly different (P = 0.9096), indicating the good predictive function of the RAREAL model. During DIXON operation for rectal cancer, the RAREAL score can be performed. If the score is more than 2.5, the possibility of postoperative AL is high.

If AL occurs after rectal cancer surgery in a patient, DS can protect the patient from complications such as sepsis. But the cost of DS is many DS related complications, the huge risk of possible DS related emergency surgery, and the decline in the quality of life after the DS. the DS should be determined according to the specific situation of each patient. Especially when new situations arise during surgery that do not match preoperative predictions, the DS or not, is sometimes a difficult option for the surgeon. The RAREAL model is a relatively simple and easy to master, established a score of 2.5 as a reference for clinicians and surgeons. If the score is more than 2.5, preventive DS should be performed to avoid severe postoperative infection. The variables used in RAREAL, which are simple and easy to use, are also familiar to clinicians, and this model facilitates surgeons to make rapid and correct judgments. This model may obviate the need for the DS in patients with a low risk of AL, helping to achieve a more rational allocation of healthcare resources, thus improving patient quality of life and reducing financial burden on patients.

Our study had the following limitations, first, the retrospective nature of our study not only made bias from patient selection and data collection difficult to avoid, but its nonrandomized study design made it impossible to control or correct for each bias in this study design. Second, the limited number of patients in the AL group in our report might hinder the discovery of more other risk factors for AL in our study.

## Conclusion

In conclusion, the RAREAL score can be used to assess the risk of AL after Dixon surgery for rectal cancer, and prophylactic DS should be proactively done when the score is greater than 2.5.

## Data Availability

The datasets used and/or analysed during the current study available from the corresponding author on reasonable request.

## Abbreviations

RAREAL: Risk assessment of rectal anastomotic leak
AL: Anastomotic leakage
NAL: Non anastomotic leakage
ALT: Intraoperative air leak test
MDT: Multidisciplinary team (MDT)
HbA1c: Glycated hemoglobin
DS: Diverting stoma
LCA: Left colic artery,
TD: Tumor distance from the anal margin
PCT: Preoperative chemoradiotherapy
ASA: American Society of Anesthesiologists physical status classification
IQR: Interquartile range
BMI: Body mass index
AUC: Area under the curve
ROC: Receiver operating characteristic
SE: Standard error

## References

[1] Olsen BC, Sakkestad ST, Pfeffer F, Karliczek A (2019). Rate of Anastomotic Leakage after rectal anastomosis depends on the definition: pelvic abscesses are significant. Scand J Surg.

[2] Wang ZJ, Liu Q. A Retrospective Study of Risk Factors for Symptomatic Anastomotic Leakage after Laparoscopic Anterior Resection of the Rectal Cancer without a Diverting Stoma. *Gastroenterol Res Pract* 2020, 2020:4863542. 10.1155/2020/4863542 PMC7174905 32351555

[3] Anderin K, Gustafsson UO, Thorell A, Nygren J (2015). The effect of diverting stoma on postoperative morbidity after low anterior resection for rectal cancer in patients treated within an ERAS program. Eur J Surg Oncol.

[4] Ihnat P, Gunkova P, Peteja M, Vavra P, Pelikan A, Zonca P (2016). Diverting ileostomy in laparoscopic rectal cancer surgery: high price of protection. Surg Endosc.

[5] Ito M, Sugito M, Fau - Kobayashi A, Kobayashi A, Fau - Nishizawa Y, Nishizawa Y, Fau - Tsunoda Y, Tsunoda Y, Fau - Saito N, Saito N. Influence of learning curve on short-term results after laparoscopic resection for rectal cancer. (1432–2218 (Electronic)). 10.1007/s00464-008-9912-1 18401643

[6] Li GX, Yan Ht Fau - Yu J, Yu J, Fau - Q, Fau - Cheng X, Cheng X. [Learning curve of laparoscopic resection for rectal cancer]. (1673–4254 (Print)). 16624777

[7] Boni L, David G, Dionigi G, Rausei S, Cassinotti E, Fingerhut A (2015). Indocyanine green-enhanced fluorescence to assess bowel perfusion during laparoscopic colorectal resection. Surg Endosc.

[8] De Nardi P, Elmore U, Maggi G, Maggiore R, Boni L, Cassinotti E, Fumagalli U, Gardani M, De Pascale S, Parise P (2019). Intraoperative angiography with indocyanine green to assess anastomosis perfusion in patients undergoing laparoscopic colorectal resection: results of a multicenter randomized controlled trial. Surg Endosc.

[9] Wu Z, van de Haar RC, Sparreboom CL, Boersema GS, Li Z, Ji J, Jeekel J, Lange JF (2016). Is the intraoperative air leak test effective in the prevention of colorectal anastomotic leakage? A systematic review and meta-analysis. Int J Colorectal Dis.

[10] Allaix ME, Lena A, Degiuli M, Arezzo A, Passera R, Mistrangelo M, Morino M (2019). Intraoperative air leak test reduces the rate of postoperative anastomotic leak: analysis of 777 laparoscopic left-sided colon resections. Surg Endosc.

[11] Rahbari NN, Weitz J, Hohenberger W, Heald RJ, Moran B, Ulrich A, Holm T, Wong WD, Tiret E, Moriya Y (2010). Definition and grading of anastomotic leakage following anterior resection of the rectum: a proposal by the International Study Group of rectal Cancer. Surgery.

[12] Kim CW, Baek SJ, Hur H, Min BS, Baik SH, Kim NK (2016). Anastomotic leakage after low anterior resection for rectal Cancer is different between minimally invasive surgery and open surgery. Ann Surg.

[13] Kim JS, Cho SY, Min BS, Kim NK (2009). Risk factors for anastomotic leakage after laparoscopic intracorporeal colorectal anastomosis with a double stapling technique. J Am Coll Surg.

[14] Alekseev M, Rybakov E, Khomyakov E, Zarodnyuk I, Shelygin Y. Intraoperative fluorescence angiography as an independent factor of Anastomotic Leakage and a Nomogram for Predicting Leak for Colorectal Anastomoses. Ann Coloproctol 2021. 10.3393/ac.2021.00171.0024 PMC9650343 34289650

[15] Watanabe T, Miyata H, Konno H, Kawai K, Ishihara S, Sunami E, Hirahara N, Wakabayashi G, Gotoh M, Mori M (2017). Prediction model for complications after low anterior resection based on data from 33,411 japanese patients included in the National Clinical Database. Surgery.

[16] Arezzo A, Migliore M, Chiaro P, Arolfo S, Filippini C, Di Cuonzo D, Cirocchi R, Morino M, Collaborators RS (2019). The REAL (REctal Anastomotic Leak) score for prediction of anastomotic leak after rectal cancer surgery. Tech Coloproctol.

[17] Zheng H, Wu Z, Wu Y, Mo S, Dai W, Liu F, Xu Y, Cai S (2019). Laparoscopic surgery may decrease the risk of clinical anastomotic leakage and a nomogram to predict anastomotic leakage after anterior resection for rectal cancer. Int J Colorectal Dis.

[18] Hoshino N, Hida K, Sakai Y, Osada S, Idani H, Sato T, Takii Y, Bando H, Shiomi A, Saito N (2018). Nomogram for predicting anastomotic leakage after low anterior resection for rectal cancer. Int J Colorectal Dis.

[19] Zarnescu EC, Zarnescu NO, Costea R. Updates of risk factors for anastomotic leakage after colorectal surgery. Diagnostics (Basel) 2021, 11(12). 10.3390/diagnostics11122382 PMC8700187 34943616

[20] Yang J, Luo Y, Tian T, Dong P, Fu Z (2022). Effects of Neoadjuvant Radiotherapy on postoperative complications in rectal Cancer: a Meta-analysis. J Oncol.

[21] Kim KJ, Choi J, Bae JH, Kim KJ, Yoo HJ, Seo JA, Kim NH, Choi KM, Baik SH, Kim SG (2021). Time to Reach Target Glycosylated Hemoglobin is Associated with long-term durable Glycemic Control and Risk of Diabetic Complications in patients with newly diagnosed type 2 diabetes Mellitus: a 6-Year observational study. Diabetes Metab J.

[22] American Diabetes A (2017). 6. Glycemic targets. Diabetes Care.

[23] Fukada M, Matsuhashi N, Takahashi T, Imai H, Tanaka Y, Yamaguchi K, Yoshida K (2019). Risk and early predictive factors of anastomotic leakage in laparoscopic low anterior resection for rectal cancer. World J Surg Oncol.

[24] Fan YC, Ning FL, Zhang CD, Dai DQ (2018). Preservation versus non-preservation of left colic artery in sigmoid and rectal cancer surgery: a meta-analysis. Int J Surg.

[25] Yang X, Ma P, Zhang X, Wei M, He Y, Gu C, Deng X, Wang Z (2019). Preservation versus non-preservation of left colic artery in colorectal cancer surgery: an updated systematic review and meta-analysis. Med (Baltim).

[26] Balciscueta Z, Uribe N, Caubet L, Lopez M, Torrijo I, Tabet J, Martin MC (2020). Impact of the number of stapler firings on anastomotic leakage in laparoscopic rectal surgery: a systematic review and meta-analysis. Tech Coloproctol.

